# Bexarotene – a novel modulator of AURKA and the primary cilium in *VHL*-deficient cells

**DOI:** 10.1242/jcs.219923

**Published:** 2018-12-14

**Authors:** Pratim Chowdhury, Reid T. Powell, Clifford Stephan, Ivan P. Uray, Tia Talley, Menuka Karki, Durga Nand Tripathi, Yong Sung Park, Michael A. Mancini, Peter Davies, Ruhee Dere

**Affiliations:** 1 Center for Precision Environmental Health, Baylor College of Medicine, Houston, TX 77030, USA; 2 Center for Translational Cancer Research, Institute of Biosciences and Technology, Texas A&M College of Medicine, Houston, TX 77030, USA; 3 Department of Clinical Oncology, University of Debrecen, Debrecen 4032, Hungary; 4 Department of Molecular and Cellular Biology, Baylor College of Medicine, Houston, TX 77030, USA

**Keywords:** Primary cilia, VHL, AURKA, Primary HTS screen, Bexarotene

## Abstract

Loss of the gene von Hippel–Lindau (*VHL*) is associated with loss of primary cilia and is causally linked to elevated levels of Aurora kinase A (AURKA). We developed an image-based high-throughput screening (HTS) assay using a dual-labeling image analysis strategy that identifies both the cilium and the basal body. By using this strategy, we screened small-molecule compounds for the targeted rescue of cilia defects associated with *VHL* deficiency with high accuracy and reproducibility. Bexarotene was identified and validated as a positive regulator of the primary cilium. Importantly, the inability of an alternative retinoid X receptor (RXR) agonist to rescue ciliogenesis, in contrast to bexarotene, suggested that multiple bexarotene-driven mechanisms were responsible for the rescue. We found that bexarotene decreased AURKA expression in *VHL*-deficient cells, thereby restoring the ability of these cells to ciliate in the absence of *VHL*. Finally, bexarotene treatment reduced the propensity of subcutaneous lesions to develop into tumors in a mouse xenograft model of renal cell carcinoma (RCC), with a concomitant decrease in activated AURKA, highlighting the potential of bexarotene treatment as an intervention strategy in the clinic to manage renal cystogenesis associated with *VHL* deficiency and elevated AURKA expression.

## Abstract (precis)

**Highlighted Article:** An image-based screen using a dual labeling strategy identified bexarotene, a rexinoid, as a novel modulator of the primary cilium in *VHL*-deficient cells.

## INTRODUCTION

Loss or mutations in the von Hippel–Lindau (*VHL*) gene are most commonly associated with VHL disease and clear cell renal cell carcinoma (ccRCC) (Kaelin, 2007). Recent advances in understanding the role of VHL in multiple molecular pathways, have resulted in the development of targeted therapies including tyrosine kinase inhibitors (TKIs), monoclonal antibodies, mammalian target of rapamycin (mTOR) inhibitors, and immune checkpoint therapy for the treatment of ccRCC (Hsieh et al., 2017). Although successful, these strategies have plateaued since their inception in large part due to the development of resistance. The molecular mechanisms underlying RCC pathogenesis are only beginning to emerge with the identification of novel targets of VHL (Anderson et al., 2011; Hasanov et al., 2017; Ivan et al., 2001; Kuznetsova et al., 2003; Lai et al., 2011b; Mikhaylova et al., 2008; Na et al., 2003; Okuda et al., 2001; Xie et al., 2009; Xue et al., 2012) linking it to a range of cellular functions including maintenance of mitotic spindle orientation (Hell et al., 2014; Thoma et al., 2009) and the primary cilium (Dere et al., 2015; Lolkema et al., 2008; Thoma et al., 2007).

VHL disease is part of a diverse group of human disorders referred to as ‘ciliopathies’, characterized by the structural loss or functional abnormality of the primary cilium, which is directly linked to the cystic kidney phenotype commonly associated with these diseases (Seeger-Nukpezah et al., 2013). The primary cilium is a microtubule-based structure that grows and resorbs in response to cues arising during the cell cycle and from environmental factors (Reiter and Leroux, 2017). Several critical regulators of cilium disassembly have been identified (Inaba et al., 2016; Inoko et al., 2012; Kim et al., 2015b; Kinzel et al., 2010; Wang et al., 2014) with Aurora kinase A (AURKA) being a key player involved in resorption of the cilium (Plotnikova et al., 2012; Pugacheva et al., 2007). We recently identified AURKA as a direct target of the E3 ligase activity of VHL (Hasanov et al., 2017), and linked elevated AURKA to the loss of primary cilia associated with *VHL* deficiency (Dere et al., 2015; Hasanov et al., 2017).

Retinoids are biologically active compounds of natural (derived from vitamin A) and synthetic origin that are intimately involved in a number of developmental and physiological processes (Bastien and Rochette-Egly, 2004; Kastner et al., 1995; Uray et al., 2016). These lipid-soluble compounds bind, as ligands, to a specific subset of nuclear receptors called retinoic acid receptors (RARs) or retinoid X receptors (RXRs) to directly regulate expression of genes involved in a diverse array of cellular functions (Allenby et al., 1993; Germain et al., 2006; Heyman et al., 1992; Levin et al., 1992). In the classical sense, receptor-mediated retinoid signaling is a function of active metabolites, their receptors and dimerization partners (Uray et al., 2016). However, studies have also demonstrated the ability of retinoids to activate several kinase cascades, suggesting that retinols could exert their non-genomic effects via extra-nuclear interactions (Aggarwal et al., 2006; Alsayed et al., 2001; Berry et al., 2012; Dey et al., 2007; Lösel and Wehling, 2003; Masiá et al., 2007; Piskunov and Rochette-Egly, 2012).

Retinoids, owing to their ability to promote cell differentiation and cell death, have been used in clinical settings for cancers including leukemia, cutaneous T-cell lymphomas, neuroblastomas, breast and lung cancers, as well as for neurological diseases and, most successfully, in treatment for dermatological disorders (Uray et al., 2016). The efficacy of retinoids in metastatic RCC was evaluated in the early 1990s with combination therapy reported to be more promising than mono-therapy for treatment of RCC (Aass et al., 2005; Berg et al., 1999; Boorjian et al., 2007; Motzer et al., 1999, 2000). Detailed evaluations revealed that all types of RAR (α, β and γ) and RXR (α and β) subtypes of receptors are expressed in RCC, although RXRγ was lost in advanced stage RCC (Lenko et al., 2013).

We have developed a primary image-based high-throughput screening (HTS) assay to identify small molecules that restore cilia in *VHL*-deficient cells. A customized image analysis algorithm was established using a dual-labeling strategy for the cilium and basal body that significantly increased accuracy and reproducibility. Bexarotene, a synthetic rexinoid was identified and validated as a bona fide regulator of primary cilia in hTERT RPE1 and mouse embryonic fibroblast (MEF) cells with an acute loss of *VHL*. In contrast to bexarotene, LG100268 (LG268), an alternative RXR agonist, failed to rescue defective cilia in *VHL*-deficient cells whereas TTNPB, an RAR agonist, only partially rescued cilia, suggesting that the rescue of cilia in *VHL*-deficient cells might be specific to bexarotene. Importantly, bexarotene-driven restoration of primary cilia in *VHL*-deficient cells correlated to a decrease in AURKA expression. Treatment of an RCC 786-0 tumor xenograft model with bexarotene showed a dramatic decrease in tumor incidence in mice treated with bexarotene compared to the vehicle-treated group, which was reflected in a tumor volume that was significantly lower in bexarotene-treated animals compared to that in vehicle controls. These data correlated to a corresponding decrease in AURKA activity in tumors from bexarotene-treated animals that did progress to fulminant tumors, indicative of the efficacy of bexarotene in modulating AURKA *in vivo*. These data establish bexarotene as a regulator of primary cilia and highlight its potential use as a clinical intervention strategy in VHL disease and other ciliopathies where AURKA expression is elevated.

## RESULTS

### Development of a primary HTS assay identifies small molecules that rescue primary cilia in *VHL*-deficient cells

**Fig. 1.**
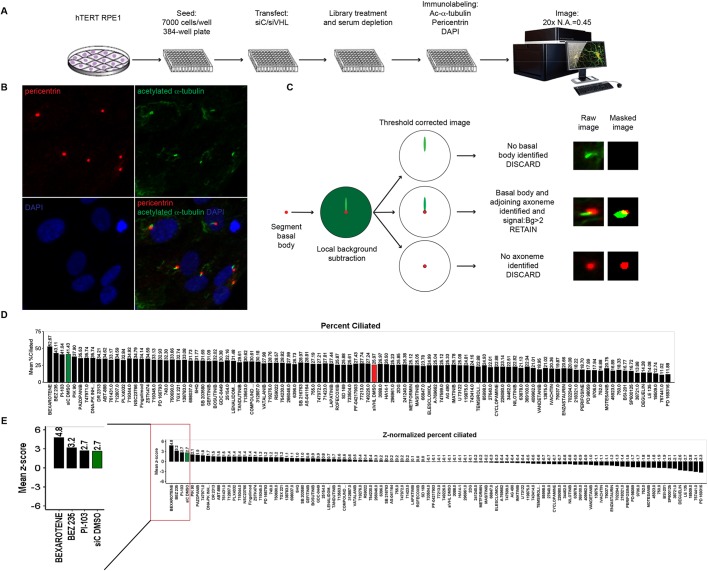
**Primary image-based HTS assay.** (A) Schematic depicting the workflow used for the development of the primary screen. (B) Representative images depicting the surface mask generated for the primary cilium (green) and the basal body (red) for further image analysis. (C) Logic used to develop the dual labeling of cilia and basal bodies for image analysis. (D) Graphical representation of data obtained following image analysis showing percentage of ciliated cells for each of the treated compounds as indicated. siVHL DMSO (vehicle), red bar; and siC (scrambled control) DMSO (vehicle); green bar. (E) Graphical representation of the *Z*-normalized percentage of ciliated cells for each of the indicated treatments. The positive hits are magnified and shown in the inset to the left. Scrambled control (siC) DMSO (vehicle), green bar.

Loss of *VHL* results in loss of primary cilia arising in part due to elevated AURKA levels (Dere et al., 2015; Hasanov et al., 2017). We developed a HTS assay to identify small molecules that could restore primary cilia in *VHL*-deficient cells, with the dual goal of identifying novel therapeutic targets and signaling pathways involved in aberrant ciliogenesis associated with loss of *VHL*. We used an *in vitro* ciliogenesis model, which we have previously established (Dere et al., 2015; Hasanov et al., 2017), wherein immortalized human retinal pigmented epithelial (hTERT-RPE1) cells transfected with VHL siRNA (siVHL, to induce an acute loss of *VHL*) were stimulated to ciliate by serum withdrawal for 48 h. We showed that this acute loss of *VHL* (siVHL) resulted in a significant decrease in the ability of hTERT RPE1 cells to ciliate compared to control siRNA (siC)-transfected cells (Dere et al., 2015; Hasanov et al., 2017). For the primary screen, the assay was re-developed to be amenable to a 384-well plate format and was performed as detailed in the schematic shown in Fig. 1A. hTERT-RPE1 cells were transfected with siC or siVHL, 24 h after seeding (7000 cells/well), and were induced to ciliate by the simultaneous withdrawal of serum and treatment with either vehicle (DMSO) or compound (detailed in Table S1) at a dose of 10 µM for 48 h. The efficiency of VHL knockdown was assessed via RT-PCR, which showed a 70–80% decrease in VHL transcript levels (shown in Fig. 4D) corroborating our previously established data (Dere et al., 2015; Hasanov et al., 2017). At the end of the incubation period (48 h), cells were immunostained for acetylated α-tubulin (a cilia marker) and pericentrin (a basal body marker) and imaged at 20× magnification (4 fields/well) using an InCell6000 confocal imaging platform.

Next, we developed a customized image analysis algorithm to score and compute the number of ciliated cells present after treatment of the *VHL*-deficient cells with vehicle or compound. We used a strategy of dual labeling of the primary cilium and the basal body, which yielded high accuracy and reproducibility as it precluded the inclusion of non-ciliary cytoplasmic acetylated α-tubulin signal (false positives for primary cilia). Using a K-mean segmentation model, a surface mask was created, for each of the cilium, basal body and nucleus (Fig. 1B). The algorithm was designed so that only signal obtained from an intersecting mask for the cilium and basal body was taken as a true signal (Fig. 1C). Since ‘hits’ identified from an HTS assay are obtained from a single well, and need to be sufficiently robust and reliable to ensure confidence in the data, we used the statistical measure of Z′, which accounts for dynamic range and signal variability. Our HTS assay yielded a Z′ value of 0.62, which is above the accepted value of 0.4 (Z′ approaching 1 is considered most desirable), and demonstrated the tractability of our image-based HTS assay. To eliminate wells in which a false signal might be generated from only a few cells that survived treatment (i.e. treatment resulted in significant toxicity), a toxicity filter (with a stringent 80% survival rate as compared to the scrambled siRNA-transfected cells) was further applied to post-acquisition image analysis, which gave us the raw mean percentage of ciliated cells (Fig. 1D). Finally, computation of Z-scores identified compounds, that is positive ‘hits’, at three standard deviations above the population mean (Fig. 1E). Two compounds, bexarotene (a synthetic rexinoid) and BEZ235 (a dual PI3K and mTOR inhibitor) were identified as potential ‘hits’ and modulators of primary cilia in *VHL*-deficient cells (Fig. 1E).

### Bexarotene is a bona fide positive regulator of primary cilia

**Fig. 2.**
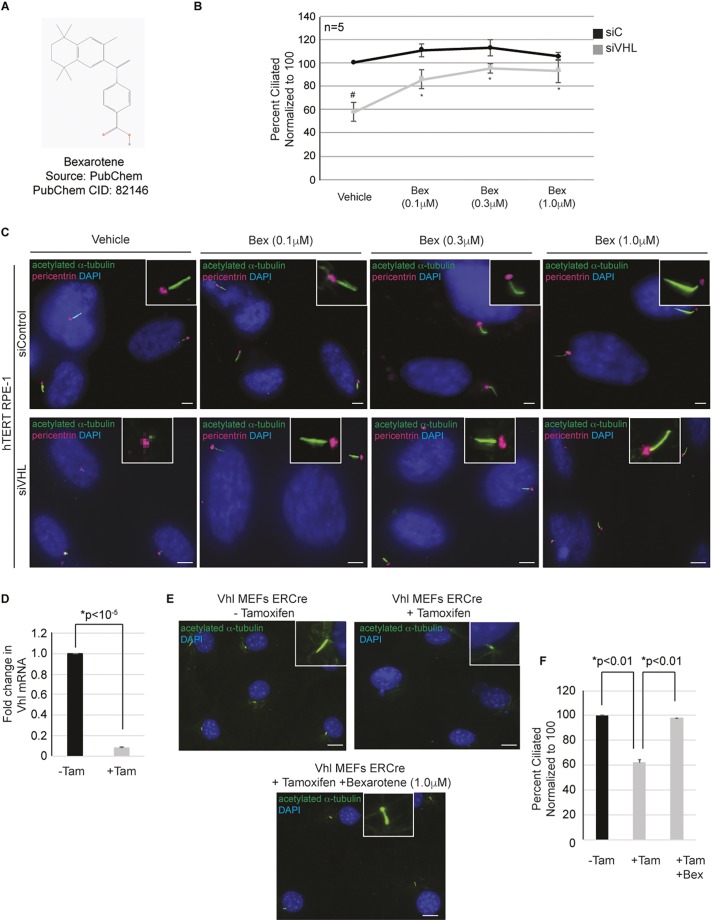
**Bexarotene dose–response curves validate its ability to restore cilia in *VHL*-deficient cells.** (A) Chemical structure of bexarotene. (B) Graphical representation of the percentage of ciliated cells normalized to the result for vehicle (set at 100%), in response to differing doses of bexarotene (Bex) in *VHL*-proficient (siC) and *VHL*-deficient (siVHL) cells. ^#^*P*<0.001 compared to siC vehicle (DMSO)-treated control; *P<0.05 denotes statistical significance compared to siVHL vehicle (DMSO)-treated control. (C) Representative images taken with a 60× objective showing primary cilia (green) and basal bodies (red). Nuclei are counterstained with DAPI in blue. Insets show a magnified image of a cilium for each of the indicated conditions. Scale bars: 5 µm (siC); 10 µm (siVHL). (D) Graph indicating the fold change in *Vhl* mRNA transcript in *Vhl^loxP/loxP^* MEF cells stably expressing a tamoxifen-inducible ERCre recombinase. Black bars denote *Vhl^loxP^^/loxP^* MEF controls, and gray bars indicate the *Vhl^loxP/loxP^* MEFs treated with tamoxifen to induce the Cre recombinase. (E) Representative images (taken with a 60× objective) from *Vhl^loxP/loxP^* MEFs controls (upper left), *Vhl^loxP/loxP^* MEFs treated with tamoxifen (upper right) and *Vhl^loxP/loxP^* MEFs treated with tamoxifen and bexarotene for 48 h (bottom center). Cells were labeled for acetylated α-tubulin (cilia marker in green) and counterstained with DAPI in blue. Insets show a magnified image of a cilium for each of the indicated conditions. Scale bars: 5 µm. (F) Graphical representation of the percentage of ciliated cells, normalized to the result for control (set at 100%; −Tam, black bar), for tamoxifen-treated (+Tam, gray bar) and tamoxifen- and bexarotene-treated (+Tam +Bex, gray bar) *Vhl^loxP/loxP^* MEFs. Results are for *n*=2 with >450 cells counted per condition. * denotes statistical significance between the groups as indicated.

Given that bexarotene (LGD1069) (Fig. 2A), yielded a mean Z-score of 4.8, we moved bexarotene into secondary validation assays to confirm its role as a positive regulator of the primary cilium. To validate bexarotene as a bona fide hit, dose–response studies were performed with freshly procured compound (two independent sources, distinct from that used for the primary assay) to assess the ability of bexarotene to rescue cilia. Dose–response curves were generated using hTERT RPE1 cells transfected with siC or siVHL, and treated with three low doses of compound (0.1 µM, 0.3 µM and 1 µM) to prevent off-target effects that might occur at the higher dose (10 µM) used for the primary screen. Image analysis using our customized algorithm established bexarotene (Fig. 2B,C) as a bona fide ‘hit’ that successfully rescued aberrant ciliogenesis in cells with VHL knockdown. We observed between a 33% to 40% increase in the number of cells that recovered their ability to ciliate in the absence of VHL (cells with a 70% knockdown of VHL) when treated with bexarotene (0.1–1.0 µM, respectively) compared to the vehicle (DMSO)-treated controls (Fig. 2B). To further validate these data, we generated *Vhl^loxP/loxP^* MEFs stably expressing a tamoxifen-inducible *ERCre* recombinase, which, when treated with 4-hydroxy tamoxifen, resulted in a 90% knockdown of *Vhl* mRNA expression (Fig. 2D). Next, to determine the effect of *Vhl* loss on primary cilia, the MEFs were maintained in serum-free medium with concurrent treatment with tamoxifen for 48 h to induce ciliation. As anticipated, we saw a statistically significant decrease in the percentage of ciliated cells under conditions of tamoxifen treatment compared to untreated cells (Fig. 2E,F). The decrease in ciliation frequency was also accompanied by the presence of shorter cilia, which is comparable to our observations in hTERT RPE1 cells with VHL knockdown (Fig. 2E). Importantly, simultaneous treatment of MEFs with tamoxifen and bexarotene (1.0 µM) in conditions of serum starvation (to induce ciliation) almost completely rescued the ability of cells to ciliate (Fig. 2E,F), further validating our data from the primary screen.

### Alternative RXR and RAR agonists fail to rescue ciliogenesis in *VHL*-deficient cells

**Fig. 3.**
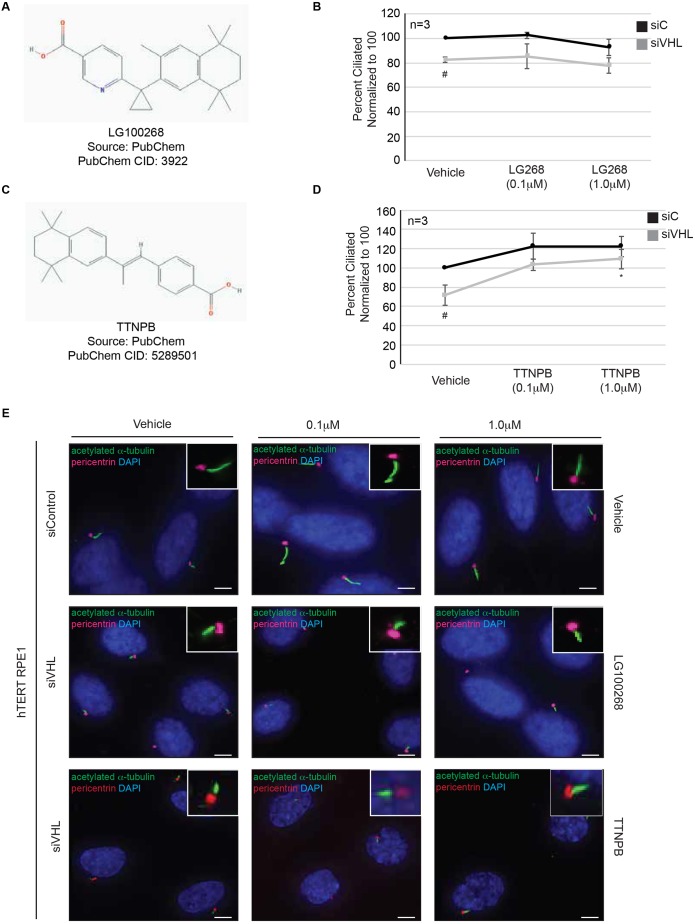
**Alternative RXR and RAR agonists fail to rescue ciliogenesis in *VHL*-deficient cells.** (A) Chemical structure of LG100268 (LG268), an RXR agonist. (B) Graphical representation of the percentage of ciliated cells normalized to the result for vehicle (set at 100%), in response to differing doses of LG268 in *VHL*-proficient (siC) and *VHL*-deficient (siVHL) cells. ^#^*P*<0.001 compared to siC vehicle (DMSO)-treated control. (C) Chemical structure of TTNPB, an RAR agonist. (D) Graphical representation of the percentage of ciliated cells normalized to the result for vehicle (set at 100%), in response to differing doses of TTNPB in *VHL*-proficient (siC) and *VHL*-deficient (siVHL) cells. ^#^*P*<0.05 compared to siC vehicle (DMSO)-treated control; *P<0.05 compared to siVHL vehicle (DMSO)-treated control. (E) Representative images taken with a 60× objective showing primary cilia (green) and basal bodies (red). Nuclei are counterstained with DAPI in blue. Insets show a magnified image of a cilium for each of the indicated conditions. Scale bars: 5 µm.

Given that bexarotene binds to and activates RXRs, we evaluated the ability of LG100268 (LG268) (Fig. 3A), an alternative rexinoid with a 10-fold-higher affinity for RXR compared to bexarotene (Liu et al., 2002), to modulate primary cilia in *VHL*-deficient cells. Surprisingly, in contrast to treatment with bexarotene, which increased the number of cells that could ciliate in the absence of VHL (Fig. 2B,F), treatment of cells with LG268 failed to rescue the ciliation defects in *VHL*-deficient cells (Fig. 3B,E). Next, to evaluate whether the effects of bexarotene could be mimicked by ligand-mediated activation of retinoic acid receptors (RARs) we used an analog of retinoic acid, TTNPB (Fig. 3C), to assess ciliation in *VHL*-deficient cells. In contrast to LG268, treatment of *VHL*-deficient cells with TTNPB only partially rescued the ability of these cells to ciliate (*P*=0.05) at the 1.0 µM dose (Fig. 3D,E) suggesting that the effects of bexarotene on primary cilia could in part be receptor mediated.

### Bexarotene rescues cilia by regulating AURKA expression

**Fig. 4.**
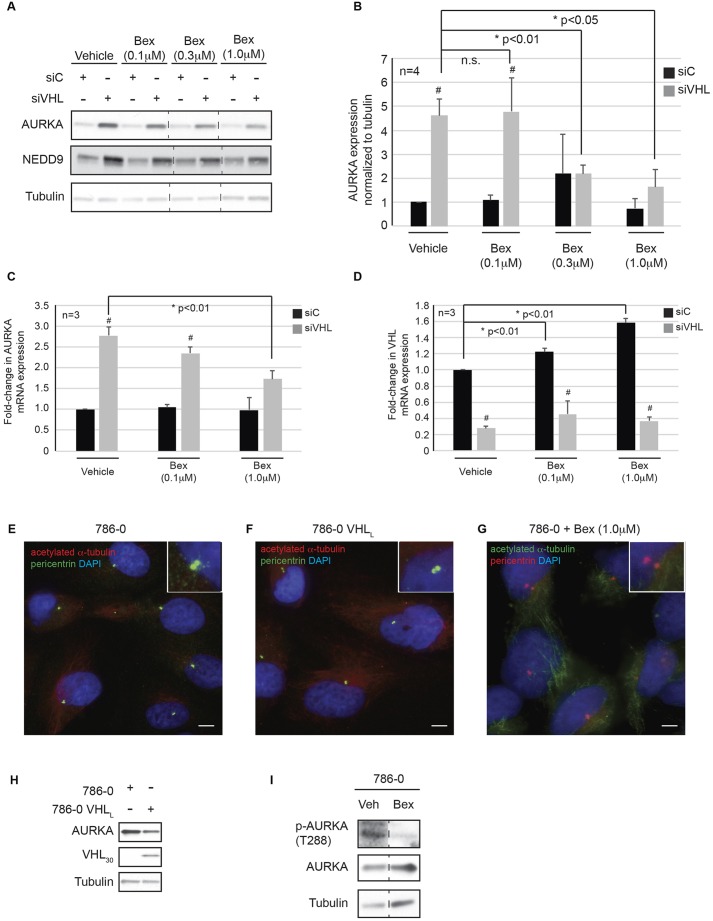
**Bexarotene regulates AURKA expression in *VHL*-deficient cells.** (A) *VHL*-proficient (siC) and *VHL*-deficient (siVHL) cells were treated with the indicated doses of bexarotene for 48 h and blotted for the antibodies as shown. Dashed lines indicate where lanes have been spliced from the same immunoblot for illustration purposes. (B) Densitometric quantitation showing the ratio of AURKA to tubulin for each of the conditions indicated. Black bars, siC; gray bars, siVHL. n.s., not statistically significant; *P-value as indicated. (C,D) RT-PCR analysis showing AURKA (C), and VHL (D) mRNA expression in *VHL*-proficient (siC, black bars) and *VHL*-deficient (siVHL, gray bars) cells in response to the indicated doses of bexarotene (serum-free medium for 48 h). (E–G) Representative images of 786-0 RCC *VHL*-null cells under the conditions indicated showing acetylated α-tubulin (cilia marker) in red, and pericentrin (basal body marker) in green. Nuclei were counterstained in blue. Insets show magnified basal bodies and cilia (cells do not present primary cilia). Scale bars: 5 µm. (H) Immunoblot analysis of lysates from 786-0 and 786-0 VHL_L_ cells blotted for the indicated antibodies. (I) Lysates from 786-0 cells treated with vehicle or bexarotene (1.0 µM) immunoblotted for the indicated antibodies.

We and others have found elevated AURKA levels to be causally linked to loss of primary cilia (Dere et al., 2015; Hasanov et al., 2017; Lolkema et al., 2008; Thoma et al., 2007) in the setting of *VHL* deficiency. This is believed to arise in part from elevated levels of AURKA, which interacts with its partner NEDD9 to activate HDAC6 and results in the destabilization of the ciliary axoneme (Pugacheva et al., 2007). To determine whether bexarotene rescued ciliogenesis by modulating AURKA expression, we initially probed AURKA levels in cellular lysates from *VHL*-deficient cells treated with bexarotene for 48 h (to induce cilia formation). We found that bexarotene decreased AURKA protein levels in a dose-dependent manner (Fig. 4A), with a parallel decrease in NEDD9 expression (AURKA binds to and stabilizes NEDD9; Pugacheva et al., 2007) (Fig. 4A). Quantification revealed a 50% reduction in AURKA levels in bexarotene-treated cells with an acute loss of *VHL* (siVHL), which decreased to match AURKA levels in bexarotene-treated control transfected (siC) cells (Fig. 4B). Given that bexarotene, in its canonical role is an RXR agonist, is known to modulate gene expression at the level of transcription (Uray et al., 2016), we next assessed *AURKA* mRNA levels in cells treated with bexarotene. Analogous to the reduction in AURKA protein expression at the 1.0 µM dose, we found a significant decrease (40%) in *AURKA* transcript levels (Fig. 4C). Importantly, bexarotene treatment also led to a dose-dependent increase in *VHL* mRNA expression in control (siC) transfected cells (Fig. 4D). However, this increase in *VHL* mRNA was suppressed in cells where VHL was knocked down by siRNA (Fig. 4D).

*VHL*-deficient RCC cell lines (786-0) lack primary cilia (Dere et al., 2015; Hasanov et al., 2017; Lolkema et al., 2008; Thoma et al., 2007); however, re-expressing VHL in these cell lines (786-0 VHL_L_) does not elicit cilia formation under the conditions used in our studies (Fig. 4E,F). Furthermore, treatment of the *VHL*-null 786-0 cells with bexarotene also failed to drive these RCC cells to ciliate (Fig. 4G). Although we do observe a decrease in AURKA levels following re-expression of VHL_L_, the inability of bexarotene to elicit a similar decrease in AURKA levels lead us to determine whether AURKA activity was modulated by bexarotene. As shown in Fig. 4I, the levels of phosphorylated AURKA decreased in 786-0 cells treated with bexarotene suggesting that bexarotene treatment might reduce AURKA levels or could effectively reduce AURKA activity.

### Bexarotene decreases tumor incidence in a mouse xenograft model of RCC

**Fig. 5.**
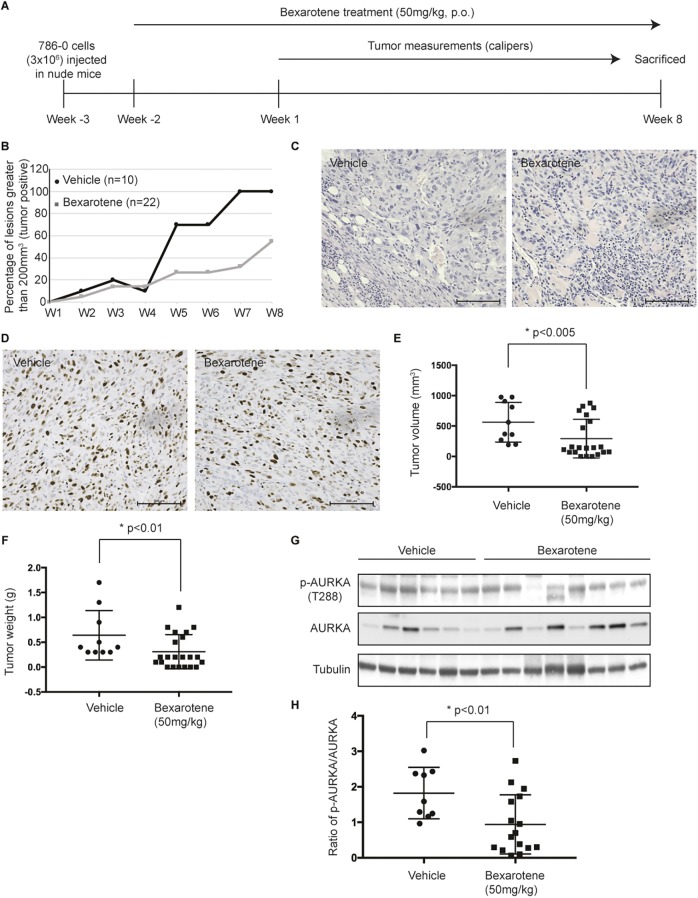
**Bexarotene decreases tumor incidence in a tumor xenograft model of RCC.** (A) Schematic depicting the workflow outlining the treatment regimen using bexarotene. (B) Rate of tumor take plotted as a percentage of lesions greater than 200 mm^3^ in size (tumor positive) at each of the indicated time points [week (W)1–W8] for vehicle (circles) and bexarotene (squares)-treated groups. (C) Representative H&E stained images (10× objective) of xenograft tumors from vehicle and bexarotene-treated animals. Scale bars: 500 µm. (D) Representative Ki67-stained images (10× objective) of xenograft tumors from vehicle- and bexarotene-treated animals. Scale bars: 500 µm. (E) Final tumor volume (mm^3^) measured following euthanasia of the mice treated with vehicle (circles) or bexarotene (squares). (F) Final tumor weight (g) measured for each tumor excised from mice treated with either vehicle (circles) or bexarotene (squares) at the time of euthanasia as shown. (G) Lysates from tumors of vehicle- and bexarotene-treated xenografts immunoblotted for the indicated antibodies. (H) Densitometric analysis showing a ratio of phospho-AURKA to total AURKA in vehicle- (circles) and bexarotene (squares)-treated xenograft tumors. * denotes statistical significance between the groups as indicated.

Given that RCC cells rarely ciliate in culture, it would be extremely challenging to evaluate rescue of cilia in a an animal model with monogenic loss of *Vhl* (which also fails to display a renal phenotype; Hsu, 2012) or a xenograft model. However, to determine whether bexarotene could affect tumor growth in *VHL*-deficient cells by modulating AURKA, we established an RCC tumor xenograft using 786-0 (*VHL*-deficient) cells in athymic nude mice (*Foxn1^nu^*). As outlined in the schematic shown in Fig. 5A, bexarotene treatment was started 2 weeks after the cells were injected in nude mice. We found that 50 mg/kg body weight/day bexarotene treatment drastically reduced tumor take to 50% of that seen in vehicle-treated mice (Table 1). Mice in the vehicle group showed a steady increase in tumor incidence, resulting in 100% of lesions/nodules reaching a size greater than 200 mm^3^ (tumor positive) (Fig. 5B; Table 1) whereas bexarotene treatment led to only 55% of the nodules reaching a size greater than 200 mm^3^. Although tumor take rates differed significantly, once the tumors presented, the growth rates of the tumors were identical in bexarotene- and vehicle-treated mice. Corroborating these data, immunohistochemistry analysis of tumor tissue using H&E and Ki67 staining failed to reveal any differences between vehicle- and bexarotene-treated animals (Fig. 5C,D). Tumor volumes (Fig. 5E) and tumor weights (Fig. 5F) measured post euthanasia (10 weeks after commencement of bexarotene treatment) were concomitantly reduced in bexarotene-treated animals compared to the vehicle controls. Although the 786-0 xenograft tumors would not form cilia, we evaluated the efficacy of bexarotene in modulating AURKA. Phosphorylation of AURKA (at T288) was evaluated in tumors from xenografts that did indeed progress to fulminant tumors (>200 mm^3^, tumor positive) in bexarotene- and vehicle-treated animals (Fig. 5G). Quantification revealed that bexarotene retained its ability to modulate AURKA activity, which was significantly reduced in bexarotene-treated xenografts compared to that seen in vehicle controls (Fig. 5H). These data suggest that bexarotene might be relevant in preventing tumor onset and reducing tumor growth.

**Table 1.**
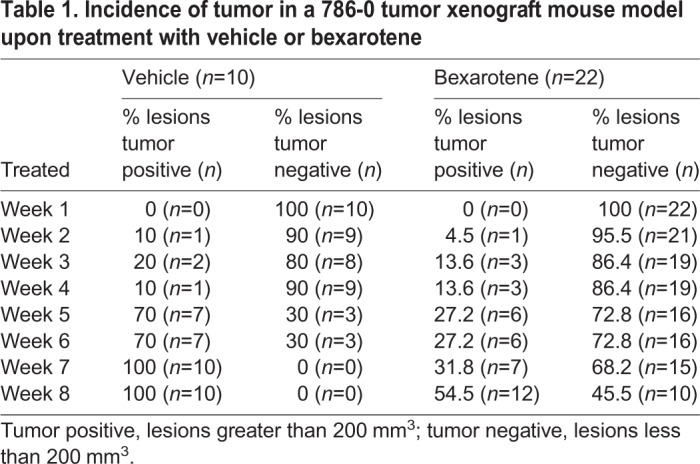
**Incidence of tumor in a 786-0 tumor xenograft mouse model upon treatment with vehicle or bexarotene**

## DISCUSSION

We have developed an image-based HTS assay, using a dual-labeling strategy for analysis, that reliably identifies both the cilium and the basal body, enabling screening of small-molecule compounds for the targeted rescue of cilia defects associated with *VHL* deficiency. A pilot screen with 146 compounds identified bexarotene as a bona fide regulator of the primary cilium. Bexarotene decreased AURKA expression and activity, providing a potential mechanism by which ciliogenesis was restored in the setting of *VHL* loss. Importantly, the inability of alternative RXR or RAR agonists to fully rescue ciliogenesis, in contrast to bexarotene, suggests that the impact on ciliogenesis might be bexarotene specific. Finally, treating RCC (786-0) tumor xenografts with bexarotene prior to tumor formation dramatically reduced tumor take (incidence) in mice compared to vehicle-treated controls. Although half the nodules in bexarotene-treated animals did not progress to fulminant tumors, the efficacy of bexarotene in modulating AURKA remained unaltered in tumors from these xenografts, highlighting the potential of exploring bexarotene as an intervention strategy in the clinic to manage renal cystogenesis associated with VHL disease and RCC.

The importance of the primary cilium is well established, although the dynamics that govern cilia formation and disassembly are only just beginning to emerge. The recent advent of large proteomic studies and bioinformatics identified thousands of proteins associated with the cilium and/or basal body referred to as the ‘ciliome’ (Gherman et al., 2006; Inglis et al., 2006). Although proteomic approaches are powerful, the lack of functional links between these candidate proteins and cilia dynamics has led to the development of targeted screens using reverse genetic approaches to specifically identify genes that modulate ciliogenesis. In addition to these global siRNA strategies (Wheway et al., 2015), key regulators of the cilium have been further identified using both screens aimed at targeting smaller subsets of therapeutically relevant genes across the genome (Kim et al., 2010; Lai et al., 2011a) and more specific screens like those evaluating the contributions of ion channels on ciliogenesis (Slaats et al., 2015). The strategy employed in our study differs from previous screens in its use of an unbiased approach, agnostic to the genes involved in ciliogenesis, to identify small-molecule compounds that can restore cilia in *VHL*-deficient cells. Use of a dual labeling scheme and increased throughput enabled the acquisition of images at 20× magnification, which further increased accuracy of image analysis and minimized false positives arising from the inclusion of cytoplasmic acetylated α-tubulin.

We identified bexarotene as a regulator of ciliogenesis in *VHL*-deficient cells. Bexarotene is a U.S. Food and Drug Administration (FDA) approved synthetic rexinoid with known function in activating RXRs (Henney, 2000). Ligand binding and activation of the cognate RXR receptors, modulates gene expression arising from the homo- or hetero-dimerization of these receptors (with RAR, PPAR, VDR and others) (Uray et al., 2016). Our observation that bexarotene modulated ciliogenesis, in contrast to an alternate RXR agonist (LG268, which has a 10-fold higher affinity for RXR compared to bexarotene; Liu et al., 2002) that failed to rescue ciliogenesis, suggests that bexarotene might act by a mechanism other than targeting RXRs, although the two may not necessarily be mutually exclusive. Recently, Src was identified as a novel ‘off-target protein’ modulated by bexarotene; *in silico* modeling studies suggested that bexarotene could inhibit Src by binding its ATP-binding pocket (Kim et al., 2015a). In addition, bexarotene has also been reported to activate p53 by post-translational modifications, namely phosphorylation on serine 15, which regulated p53 downstream targets such as p21, and could also modulate AURKA both at the transcript and protein level (Nieto-Rementería et al., 2009). Our data demonstrate the ability of bexarotene to modulate AURKA mRNA expression only at the 1.0 µM dose but not at the lower doses that impact AURKA protein levels. It will now be interesting to determine whether our data identifying bexarotene as a modulator of AURKA expression in *VHL*-deficient cells provides yet another ‘off-target protein’ that might be modulated by bexarotene in an RXR-independent manner.

AURKA, in a non-mitotic role was identified as a regulator of cilium disassembly. Elevated AURKA activity resulted in activation of HDAC6, a downstream target, leading to the destabilization of the microtubule-based axoneme and cilia resorption (Pugacheva et al., 2007). We and others have shown AURKA is elevated in *VHL*-deficient RCC (Dere et al., 2015; Hasanov et al., 2017; Lolkema et al., 2008; Thoma et al., 2007), and our recent finding identified AURKA as a target of E3 ligase activity of VHL (Hasanov et al., 2017), providing a direct link between elevated AURKA and *VHL*-null RCC. Importantly, although bexarotene modulated AURKA transcript levels specifically at the 1.0 µM dose, it significantly increased *VHL* mRNA expression at all doses. Although, rexinoids have not been explored in the context of RCC, a previous study reported that a combination of 13-cis retinoic acid and an HDAC inhibitor augmented VHL expression in cells (Tavares et al., 2008). This is especially important given the recent efforts in the field to re-functionalize VHL using proteasome inhibition, which proved effective in increasing mutant VHL levels (Ding et al., 2014). Mutant VHL is degraded prematurely due to misfolding and an imbalance of chaperonin, although it still retains its E3 ligase function (Lee et al., 2009; Rechsteiner et al., 2011). Increasing VHL expression using bexarotene could be an exciting and novel approach in the clinic, especially if combined with the modulation of proteostasis.

Retinoids promote differentiation and apoptosis in cells, and this attribute has led to their successful therapeutic application in several cancers including acute promyelocytic leukemia (APL), Kaposi's sarcoma, squamous cell skin cancer, cutaneous T-cell lymphoma, neuroblastoma, lung cancer and breast cancer (Uray et al., 2016). In contrast to retinoids, treatment with rexinoids shows reduced teratogenicity and toxicity with manageable side-effects, such as hyperlipidemia (Assaf et al., 2006). The effects of retinoids in RCC treatment were deemed more efficacious when used as part of a combination approach compared to the anti-tumor activity of single agents (Aass et al., 2005; Boorjian et al., 2007; Motzer et al., 1999, 2000). These data showing limited efficacy of retinoids in RCC, led to recent efforts refocusing on identifying receptor subtypes associated with RCC and their prognostic significance (Buentig et al., 2004; Goelden et al., 2005; Obara et al., 2007). Our findings show the ability of a rexinoid, bexarotene, to reduce tumor take and decrease tumor growth in a tumor xenograft model of RCC. These data are promising as they lay the foundation for unexplored intervention strategies, focused on prevention, in high-risk VHL patients with elevated AURKA expression. Bexarotene as a prevention strategy (either singly or in combination) has found efficacy in cancers such as lung cancer (Wang et al., 2006) and oral cavity carcinogenesis (Tang et al., 2014). In the case of VHL disease and RCC, it would be valuable to assess the synergistic effects of bexarotene with anti-angiogenic drugs, and with AURKA inhibitors that can rescue the ciliation defects associated with *VHL* deficiency (Dere et al., 2015; Hasanov et al., 2017). The identification of bexarotene as a modulator of the cilium now opens new avenues to investigate the merit of rexinoids as a treatment strategy for ciliopathies.

## MATERIALS AND METHODS

### Cell culture and drug treatments

Immortalized retinal pigmented epithelial (hTERT RPE1) cells (a gift from Gregory Pazour, University of Massachusetts Medical School, Worcester, MA) were maintained in Dulbecco's modified Eagle's medium/F-12 (Life Technologies, Carlsbad, CA). The human 786-0 *VHL*-deficient RCC cell line was maintained in RPMI-1640 medium (Life Technologies). Cells were maintained in media supplemented with 10% fetal bovine serum (Sigma-Aldrich, St Louis, MO). All human cell lines were short tandem repeats fingerprinted and validated using the Characterized Cell Line Core Facility (U.T. M.D. Anderson Cancer Center). In addition, all cells used in these studies were routinely tested and confirmed negative for mycoplasma.

### Generation of *Vhl^loxP/loxP^* mouse embryonic fibroblasts

*Vhl^loxP/loxP^* MEFs were generated from 13.5 days post-coitum (d.p.c.) embryos of *Vhl^loxP/loxP^* mice (JAX Labs). These MEF cells were immortalized by serial passaging in high glucose DMEM (Life Technologies) supplemented with 10% FBS (Sigma-Aldrich) and penicillin-streptomycin (Life Technologies). Immortalized MEF cells were transfected with a tamoxifen-inducible *ER-Cre* vector expressing Cre recombinase fused with a mutated ligand-binding domain for the human estrogen receptor (ER-Cre). Stable MEF cell lines expressing ER-Cre were generated by culturing in selection media containing 5 μg/ml blasticidin (Thermo Fisher Scientific, Waltham, MA). *Vhl^flox/flox^* MEFs were treated with 3 μM 4-hydroxytamoxifen (Sigma-Aldrich) for 2 days for efficient *Vhl* knockout.

Compounds used for the primary screen (Table S1) were solubilized in DMSO and used at a final concentration of 10 µM. Bexarotene, was obtained from Sigma-Aldrich and Selleck Chem (Houston, TX); LG100268 and TTNPB were obtained from Sigma-Aldrich and used in the secondary validation studies *in vitro* at final concentrations of 0.1 µM, 0.3 µM and 1.0 µM doses (solubilized in DMSO) for 48 h as indicated for each individual treatment.

### Primary screen and image analysis

hTERT RPE1 cells (7000 cells) were plated in each well of 384 well plates and transfected with siC (catalog no. 001810-10-20, Dharmacon, Lafayette, CO) or siVHL (catalog no. L-003936-00-0005, Dharmacon). Next, cells were simultaneously treated with the compound library (Custom Clinical library with 146 compounds, Table S1) and serum starved for 48 h to induce primary cilia. Cells were subsequently fixed and stained for primary cilia, basal body and nuclei (as described below). Images were obtained at 20× magnification, four fields per well and 13 *z*-sections (0.5 µM steps) using an InCell6000 confocal microscope (GE Healthcare Life Sciences, Pittsburgh, PA).

Images were analyzed using Pipeline Pilot (BIOVIA, San Diego, CA) using an algorithm customized to measure primary cilia. First, images were subjected to a background subtraction using either Rolling Ball (set the ball radius) or Mode (subtraction of the most often pixel, i.e. black space) methods. Background-corrected images were then passed into basal body segmentation, which segmented basal body using size and a simple auto-threshold to convert the signal into a binary mask, which was further refined using a user-defined assay optimized radius (∼50 pixels). Upper and lower size filters were then applied to remove aggregates from the masked image. Next, the protocol segmented primary cilia based on the acetylated α-tubulin (acTub) channel. This was one of the trickier and elaborate parts of the protocol because it involved identifying a small subpopulation of the total acTub. Here, the acTub channel was first subjected to an aggressive linear contrast enhancement applied to the selected field which set 1% of the labeling intensity to saturation and 98% to the main. Although this resulted in essentially a binary image, there was still some noise associate with the signal. To resolve this issue a local background subtraction under the local basal body mask was applied. This was performed by constructing a normalization mask which was then multiplied by the original contrasted image. Once normalized, the pixel intensities below the 80th percentile under the local basal body masks were removed. A cluster-based segmentation was then applied to the now normalized background subtracted image. This approach helped identify objects and divide them into three intensity ranges: high, medium and background. Medium intensity regions were removed, resulting in only the high intensity regions being selected. It was important to use a three-category approach here because sometimes the cilia presided over a complex network of other signals, which would be picked up if using a binary segmentation approach. A primary touching object filter was next applied to ensure that only axonemes (a microtubule extension) touching the basal body are considered as potential primary cilia. Statistical filtering involved a user-defined size exclusion filter, and a ratio filter was applied to remove objects that took up the entire mask, which was the case with mitotic-organizing centers. A signal-to-background ratio was used for filtering, which used the mean pixel intensity of the axoneme mask to the local background (this filter used the original acTub image). The final filter used the condition that candidate primary cilia must be outside of two standard deviations from the mean pixel intensity under the local centrosome mask. Object labels that contained both a basal body and cilia label were then assigned a Boolean value of one; if the basal body alone was defined, a Boolean value of zero was assigned. The mean of the Boolean value was taken to calculate the percentage of ciliated cells.

Using the values from the segmentation protocols described above, a Z′ value was calculated using siC (scrambled control) DMSO-treated wells, as a positive control, and siVHL treated with sulforaphane (sulphoraphane causes loss of primary cilia; P.C. and R.D., unpublished data) as negative controls. Using these we obtained a Z′ factor of ∼0.6, indicating that we had developed a robust assay. To identify hits from the plates a *Z*-score was calculated using the mean and standard deviation compared to the siVHL DMSO-treated wells. Partially toxic wells were excluded on the criteria that the cell density was lower than 80% of the mean cell count in the siC DMSO-treated wells. Active wells were selected as wells that were not filtered out by the toxicity filter and were outside of three standard deviations of the value for siVHL DMSO-treated wells.

### Immunocytochemistry

Immunofluorescence staining to observe primary cilia was performed as published previously (Dere et al., 2015; Hasanov et al., 2017). Briefly, hTERT RPE-1 cells or MEFs plated on glass coverslips (secondary validation) were transfected, starved and treated for 48 h to induce cilia formation (and in the case of MEFs were accompanied by 3 μM 4-hydroxytamoxifen; Sigma-Aldrich). Cells were fixed using 4% paraformaldehyde (15 min), permeabilized with 0.5% Triton-X (10 min), followed by blocking in 3.75% bovine serum albumin solution (1 h). Primary antibodies for acetylated α-tubulin (clone 6-11B-1, 1:5000; Sigma-Aldrich) and pericentrin (1:5000; Abcam, Cambridge, MA) were applied in blocking buffer for 1 h. AlexaFluor 488 and 546 goat anti-mouse-IgG or anti-rabbit-IgG secondary antibodies (Life Technologies) were subsequently applied for another hour. Cells were counterstained using DAPI (1:4000 of 1 mg/ml stock, Thermo Fisher Scientific, Waltham, MA) and visualized using a Deltavision deconvolution microscope (Applied Precision, Pittsburgh, PA) or a Nikon Ti2 inverted microscope (Nikon, Melville, NY) at 60× magnification (secondary validation assays).

### Immunoblotting

Whole-cell lysates were collected in cold 1× cell lysis buffer (20 mM Tris-HCl pH 7.5, 150 mM NaCl, 1 mM EDTA, 1 mM EGTA, 1% Triton-X-100, 2.5 mM sodium pyrophosphate) containing 1× complete protease inhibitor (Roche, Mannheim, Germany), and 1 mM sodium orthovanadate (Na_3_VO_4_). The following primary antibodies were used for immunoblotting: anti-AURKA (cat. no. 4718S, 1:1000), anti-phospho-AURKA (cat. no. 3079S, 1:200), anti-NEDD9 (cat. no. 4044S, 1:1000) and anti-HDAC6 (cat. no. 7612S, 1:1000) from Cell Signaling Technologies (Danvers, MA); anti-tubulin (DM1A, 1:10,000) from Santa Cruz Biotechnology (Dallas, TX). Horseradish peroxidase-conjugated goat anti-mouse-IgG and goat anti-rabbit-IgG secondary antibodies were also purchased from Santa Cruz Biotechnology. Immunoblots were visualized using Pierce ECL (Thermo Fisher Scientific) substrate.

### RT-PCR analysis

Reverse transcriptase-PCR analysis was performed as described previously (Dere et al., 2015; Hasanov et al., 2017). Briefly, mRNA was isolated from cells transfected with siControl/siVHL, and cDNA was prepared by reverse transcription (Superscript III, Life Technologies). Gene expression was assessed by real-time quantitative PCR using specific TaqMan probes (Thermo Fisher Scientific, Waltham, MA), and a TaqMan Fast Universal master mix on a Viia7 system (Thermo Fisher Scientific, Waltham, MA). mRNA expression was evaluated for VHL, AURKA, GLI1, PTCH and PPIA (endogenous control). The following set of conditions were used for each real-time reaction: 95°C for 20 min followed by 40 cycles of 1 s at 95°C and 20 s at 60°C. The real-time PCR reactions were all performed in triplicate and were quantified using the −ΔΔ cycle threshold (CT) method.

### 786-0 tumor xenograft mouse model

In order to establish a RCC tumor xenograft model, athymic, immunodeficient mice (*Foxn1^nu^*, JAX Labs, Bar Harbor, ME, USA), at 5–6 weeks of age, were injected with 786-0 cells (3×10^6^ cells/0.1 ml) subcutaneously on the right thigh and scruff of each mouse. The mice were randomly divided into two groups, the vehicle (corn oil) control group (*n*=5, two sites on each mouse, 10 lesions) and the bexarotene treatment group (*n*=11, two sites on each mouse, 22 lesions) and were gavaged orally with bexarotene at a dose of 50 mg/kg body weight/day. Tumors were measured once weekly using digital calipers two weeks after commencement of drug treatment. Animals were killed 10 weeks following bexarotene treatment and tumors were re-measured to get final tumor weights and volumes. For tumor volume, the major and minor axes of the tumor were measured, and volume calculated using the formula; length×width×width/2. Specifically, for tumor take, only lesions greater than 200 mm^3^ were considered to be bona fide tumors (tumor present). Tumor tissue harvested at the time of killing was subsequently fixed in formalin and stained for H&E and Ki67 and imaged on a Nikon Ci-E microscope (Nikon, Melville, NY). All animal studies were performed in compliance with Institutional IACUC approval.

### Statistics

Densitometric analyses were performed using ImageQuant (GE Healthcare Life Sciences, Pittsburgh, PA). Significance for the *in vitro* data (densitometry and RT-PCR analyses) was determined by using Student's *t*-tests (two-tailed, assuming equal variance) from at least three biological replicates. Comparisons between groups for the animal studies were performed using Kilmogorov–Smirnov D or Mann–Whitney tests. The standard error of mean (s.e.m.) was calculated, and reported *P*-values less than 0.05 were considered to be significant. All statistics were performed using Prism 7 (GraphPad, La Jolla, CA) and Excel (Microsoft, Redmond, WA) software.

## Supplementary Material

Supplementary information
