# Supplementary material for: Bexarotene – a novel modulator of AURKA and the primary cilium in VHL-deficient cells
Source: J Cell Sci. 2018 Dec 14;131(24):jcs219923. doi: 10.1242/jcs.219923 (PMC6307881; doi:10.1242/jcs.219923)
Supplement: Supplementary information [file joces-131-219923-s1.pdf]

Table S1. Custom Clinial Library

Click here to Download Table S1
